# A shortfin mako shark circling a finless porpoise with damaged caudal fin

**DOI:** 10.1002/ece3.70024

**Published:** 2024-07-17

**Authors:** Taro Okamura, Soma Tokunaga, Takaya Ogawa, Ken Yoda

**Affiliations:** ^1^ Graduate School of Environmental Studies Nagoya University Nagoya Aichi Japan; ^2^ Department of Evolutionary Studies of Biosystems The Graduate University for Advanced Studies, SOKENDAI Hayama Kanagawa Japan; ^3^ Sport Fishing Boat WADATSUMI Captain Hatsukaichi Hiroshima Japan

**Keywords:** finless porpoise, predator–prey interaction, Seto Inland Sea, shortfin mako shark

## Abstract

Research on predator–prey interactions between sharks and cetaceans remain limited. Here, we report on a video of a shortfin mako shark circling a finless porpoise with a damaged caudal fin in the Seto Inland Sea, Japan. The finless porpoise was neither emaciated nor inactive, but unable to swim effectively due to the complete lack of a caudal fin. Some circumstantial evidence, including a bite mark on the porpoise's head, strongly suggests that the mako shark attacked it. Furthermore, the possible time difference between the two injuries the porpoise sustained may reflect the shark's hunting tactics. While mako sharks primarily feed on small fish and cephalopods, this observation suggests they also may prey on live cetaceans more often than previously thought.

## Graphical abstract

We report a video documentation of a shortfin mako shark circling a finless porpoise with a damaged caudal fin in the Seto Inland Sea, Japan. The wounds on the porpoise suggest that the shark attacked it while it was alive. While mako sharks primarily feed on small fish and cephalopods, this observation suggests they also may prey on live cetaceans more often than previously thought.

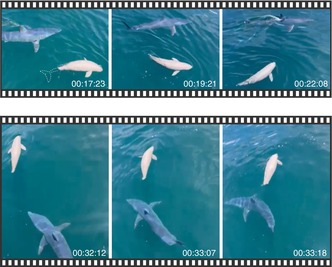

Small cetaceans (i.e., dolphins and porpoises) are often regarded as apex predators but are threatened by natural predators such as large sharks and killer whales (e.g., Heithaus, 2001; Kiszka et al., 2022). Evidence for large sharks as potential predators includes the occasional presence of small cetaceans in their stomachs (Heithaus, 2001) and shark bite marks on live cetaceans (Heithaus et al., 2017; Smith et al., 2018). Based on behavioral observations and stomach contents, Heithaus (2001) identified five shark species as regular predators: the white shark *Carcharodon carcharias*, the tiger shark *Galeocerdo cuvier*, the bull shark *Carcharhinus leucas*, the sixgill shark *Hexanchus griseus*, and the sevengill shark *Notorynchus cepedianus*. However, direct observations of shark predation events on live cetaceans are still limited, particularly for species considered occasional or suspected predators. Consequently, the predator–prey interactions between sharks and cetaceans remain unclear.

In this note, we video‐documented a shortfin mako shark *Isurus oxyrinchus* (hereafter, mako shark) circling a finless porpoise *Neophocaena asiaeorientalis* with a damaged caudal fin at the water surface. The mako shark, a large‐apex predator found in tropical and temperate waters worldwide, is considered as a suspected predator of cetaceans (Heithaus, 2001). They mainly feed on small fish and squids (Preti et al., 2012), using their smooth, slender teeth, which are suited for piercing small prey (Motta & Huber, 2012). Stomach content analyses have sometimes revealed small cetaceans, including a fresh dolphin carcass, suggesting they are capable of capturing live cetaceans (Cliff et al., 1990; Monteiro et al., 2006; Porsmoguer et al., 2015). However, direct observations of their predatory behaviors on live cetaceans are few (Leatherwood et al., 1973). The finless porpoise is a small cetacean that inhabits the nearshore areas of East Asia (Amano, 2017). Although the distribution overlaps with that of mako sharks, including in those the Seto Inland Sea of Japan, where the video was recorded, interactions with mako sharks have never been reported to our knowledge.

On August 23, 2023, at 12:25 PM, one of the authors (T. Ogawa), who is the captain of a local sport fishing boat, observed a shark swimming around a porpoise at the surface off the southeast coast of Suo Oshima in the Seto Inland Sea of Japan (33°53′54.72″ N 132°26′44.7″ E). He recorded this behavioral phenomenon in a 35‐s video using an iPhone 12 Pro (Apple Inc.) from a small boat (see Supporting Information for the full video). The resolution was 1280 × 720 pixels, with a frame rate of 30 frames/s. The shark was identified as the shortfin mako shark based on its streamlined body, long pointed snout, semifalcate pectoral fins shorter than the head, and lunate caudal fin (Ebert et al., 2021). The porpoise was identified as the finless porpoise because of its plain white body coloration, round head lacking a rostrum, large flippers with a high aspect ratio, and absence of a distinct dorsal fin (Amano, 2017; Jefferson et al., 1993).

The finless porpoise, while still able to breathe and maintain a body balance at the surface, could not escape from the shark because of severe damage to its caudal region (Figure 1; Video S1). The caudal wound was an amputation that was roughly severed by the caudal peduncle. It had already stopped bleeding, but there was no evidence of healing, such as muscular ridges on the amputation surface (Figure 2a). Another wound was observed on the head, which was located dorsally between blowhole and flipper, comprised a series of bleeding crescent‐shaped sting wounds (Figure 2c). The body of finless porpoise was not attached to any artifacts, including fishing gear, and showed no abrasions or lacerations caused by such equipment. The porpoise did not appear emaciated, judging from the lack of a distinct neck constriction, spinous processes, and transverse processes, which is caused by the loss of muscle and fat (Joblon et al., 2014). The mako shark continuously circled the porpoise and approached it twice from the caudal side (Figure 1). The porpoise reacted to the approaching shark by changing direction, which involved twisting its body and moving its flippers. No physical contact, such as biting, was observed until the end of the observation. After the observation for less than 10 min, the boat was moved away from the site without tracking the animals. Since then, there have been no further sightings of the “flukeless” porpoise in this area.

**FIGURE 1:**
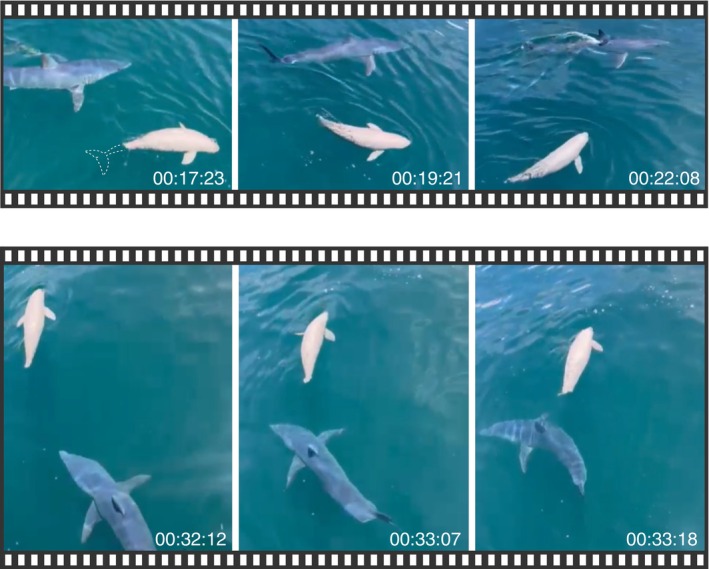
Shortfin mako shark *Isurus oxyrinchus* approaching a finless porpoise *Neophocaena asiaeorientalis* with a damaged caudal fin. The first and second approaching behaviors over time are shown in the upper and lower parts, respectively. The dashed line traced the lost region of the porpoise. For details, see Video S1. Photo credit: Takaya Ogawa.

**FIGURE 2:**
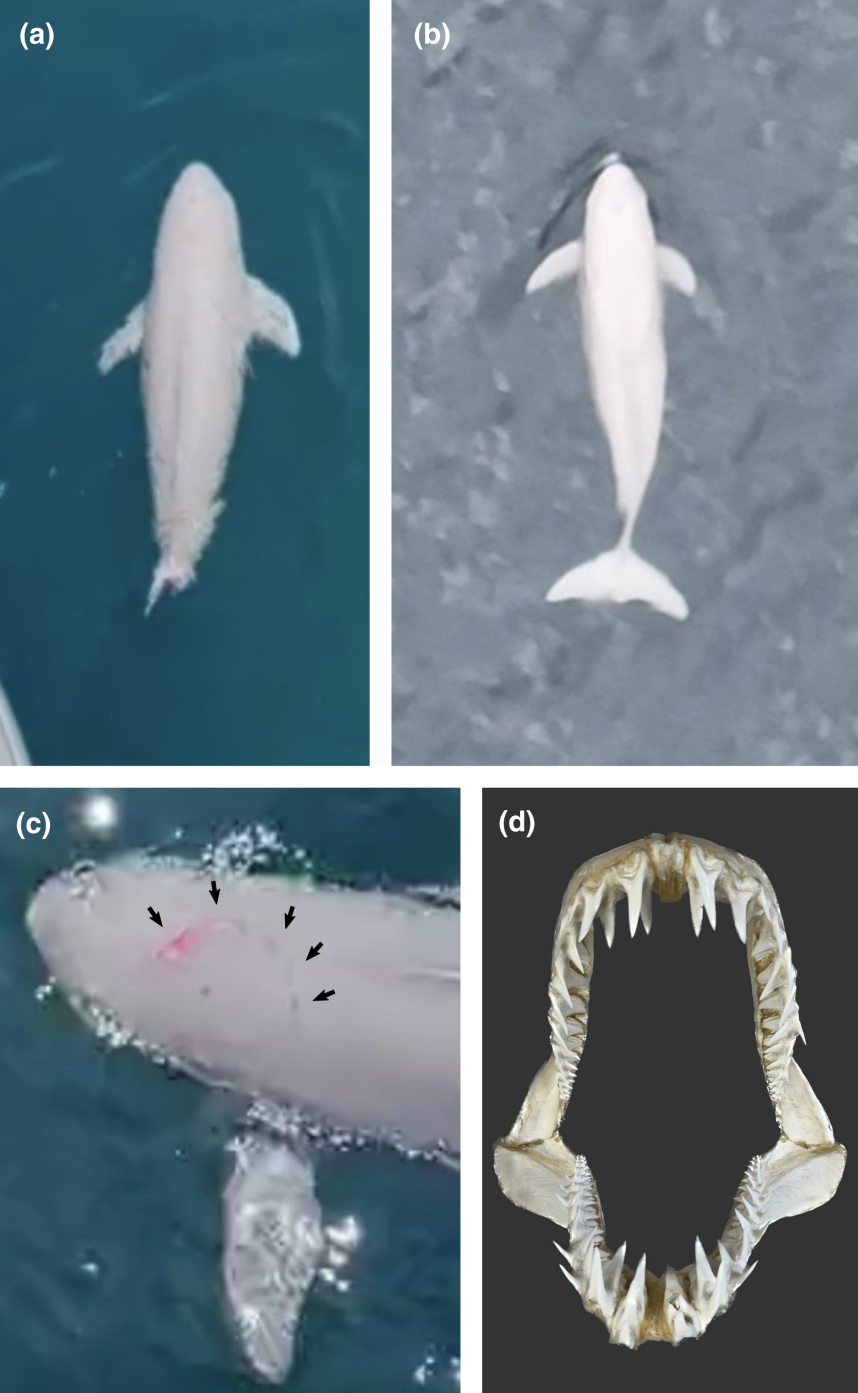
Graphic information regarding the wounds on finless porpoise. The caudal wound (a) is a rough amputation (see main text). The individual recorded in this note showed that its entire caudal fin was lacking when compared to that of healthy individuals recorded on different days in the same area (b). The head wound (c) was a series of crescent‐shaped sting wounds as indicated by the arrow, which closely resembled the teeth of shortfin mako shark (d). Photo credit: (a, c) Takaya Ogawa, (b) Satoko Kimura, Mayu Ogawa, Takehiro Ikeda, (d) Rui Matsumoto.

Circling and approaching behavior suggest that the mako shark recognized the finless porpoise and possibly prevented it from escaping. The finless porpoise seemed to move its body to escape the shark rather than to avoid sinking, but it could not swim effectively due to the lack of a caudal fin. The porpoise was not emaciated, strongly suggesting it was a recent injury. Unlike the finless porpoise shown in our video, some cetaceans can compensate for the loss of the caudal fin through behavioral adjustments. For example, a flukeless juvenile Indo‐Pacific bottlenose dolphin *Tursiops aduncus* used a unique swimming style to twist its peduncle, enabling it to survive for over 2 years (Kim et al., 2022). However, it is unlikely that such a unique swimming style was acquired immediately after severing the caudal fin. Moreover, such behavioral adaptations may develop over time, possibly under the protection of a group. In contrast, the finless porpoise, which typically does not form groups and remains solitary throughout the year in the Seto Inland Sea (Kasuya & Kureha, 1979; Shirakihara et al., 2007), may find it harder to survive long‐term without a caudal fin, unlike cetaceans living in groups.

Although direct evidence is lacking, we believe that the mako shark injured the finless porpoise based on circumstantial evidence. First, the crescent‐shaped series of sting wounds on the head was clearly consistent with shark bite marks (Heithaus et al., 2017; Smith et al., 2018), especially resembling the smooth, slender teeth of mako sharks (Figure 2d). Given the absence of other possible predators around the two animals, it is reasonable to conclude that the mako shark had bitten the head of the porpoise.

Second, the rough cross‐section of the caudal peduncle indicates that it was not severed by a ship's propeller, which would have caused sharp, multiple cuts (Byard et al., 2013; Figure 2a,b). There were no signs of disease around the caudal region, ruling out the possibility of necrosis. Moreover, it is unlikely that fishing gear caused the amputation. The non‐emaciated state of the porpoise, the presence of tissue fragments on the amputation surface, and the lack of healing consistently suggest that the injury occurred recently (i.e., minutes to hours previously) within a short time period. No fishing gear was found entangled on the body, nor were there abrasions typically caused by such equipment. The severing of the caudal region, including the vertebrae, indicates considerable force. To our knowledge, there are no direct observations of small cetaceans being amputated, including the bones, in a single contact and short time period (e.g., Nicholson, 2023; Slooten et al., 2013; Wells et al., 2008). Taken together, natural predators such as killer whales and sharks, rather than artificial or diseased factors, likely were responsible. Although killer whales may leave porpoises they attack without eating them (Giles et al., 2024), there were no sightings of a pod of killer whales in the Seto Inland Sea during the period of this observation. Considering that only a short time had passed since the finless porpoise lost its caudal fin, it is more plausible that the mako shark injured the porpoise, rather than other sharks, such as white sharks, attacking and then ignoring the immobilized porpoise. The capability of mako sharks to sever cetacean fins or bodies has been demonstrated by observations of severed dorsal fins of small dolphins (Porsmoguer et al., 2015) and a fresh dolphin carcass divided into three pieces by one bite (Monteiro et al., 2006) in the stomachs of shortfin makos. These circumstantial evidence strongly suggest that the mako shark attacked the finless porpoise. While mako sharks primarily feed on small fish and cephalopods, this observation suggests they also may prey on live cetaceans more often than previously thought.

Furthermore, different wound conditions may provide insights into the hunting tactics of mako sharks. Notably, no bleeding was observed from the severed caudal peduncle of the porpoise, despite the presence of large blood vessels (Rommel et al., 2006). Because the bleeding bite mark on the head seemed to be a fresh wound, the caudal region likely was severed first. Assuming that the mako shark inflicted both wounds, this implies that the shark may have waited after severing the caudal region of the porpoise. To bite the caudal region of cetaceans is effective in immobilizing them and thus preventing them from escaping, as previously reported for white and tiger sharks (Dines & Gennari, 2020; Maldini, 2003) and demonstrated in our video. Furthermore, white sharks occasionally await large prey (e.g., marine mammals) to weaken after inflicting serious wounds, probably to minimize the risk and energetic cost of hunting (Dines & Gennari, 2020; Tricas & McCosker, 1984). This tactic should be particularly beneficial when the size difference between the predator and prey is small, as with the mako shark and finless porpoise. Since the porpoise was still moving after losing its caudal fin, the mako shark may have waited between attacks. Unfortunately, our video lacked direct evidence for this scenario, and further research is needed to better understand the hunting tactics of mako sharks.

## AUTHOR CONTRIBUTIONS

**Taro Okamura:** Conceptualization (lead); formal analysis (lead); visualization (lead); writing – original draft (lead); writing – review and editing (equal). **Soma Tokunaga:** Conceptualization (lead); formal analysis (lead); writing – original draft (lead); writing – review and editing (equal). **Takaya Ogawa:** Data curation (lead); investigation (lead); resources (lead); writing – review and editing (equal). **Ken Yoda:** Funding acquisition (lead); project administration (lead); supervision (lead); writing – review and editing (supporting).

## FUNDING INFORMATION

This work was supported by the JSPS KAKENHI (Grant Number JP21H05294). This work was also supported by JSPS Research Fellowship for Young Scientists to T. Okamura and S. Tokunaga. Open Access funding provided by Nagoya University.

## CONFLICT OF INTEREST STATEMENT

The authors have no conflict of interests to declare that are relevant to the content of this article.

## Supporting information


Video S1

## Data Availability

The video data that underpin the conclusions has been uploaded as Supporting Information.
