# Supplementary material for: A shortfin mako shark circling a finless porpoise with damaged caudal fin
Source: Ecol Evol. 2024 Jul 17;14(7):e70024. doi: 10.1002/ece3.70024 (PMC11255379; doi:10.1002/ece3.70024)
Supplement: Supplementary file 1 — Video S1 [file ECE3-14-e70024-s001.zip › VideoS1_Legends.docx]

Video S1: Shortfin mako shark approaching a finless porpoise with a damaged caudal fin. The video was taken by chance by one of the authors. Video credit: Takaya Ogawa.
